# Supplementary figures and images for: Epidemiological insights from a large-scale investigation of intestinal helminths in Medieval Europe
Source: PLoS Negl Trop Dis. 2020 Aug 27;14(8):e0008600. doi: 10.1371/journal.pntd.0008600 (PMC7451528; doi:10.1371/journal.pntd.0008600)

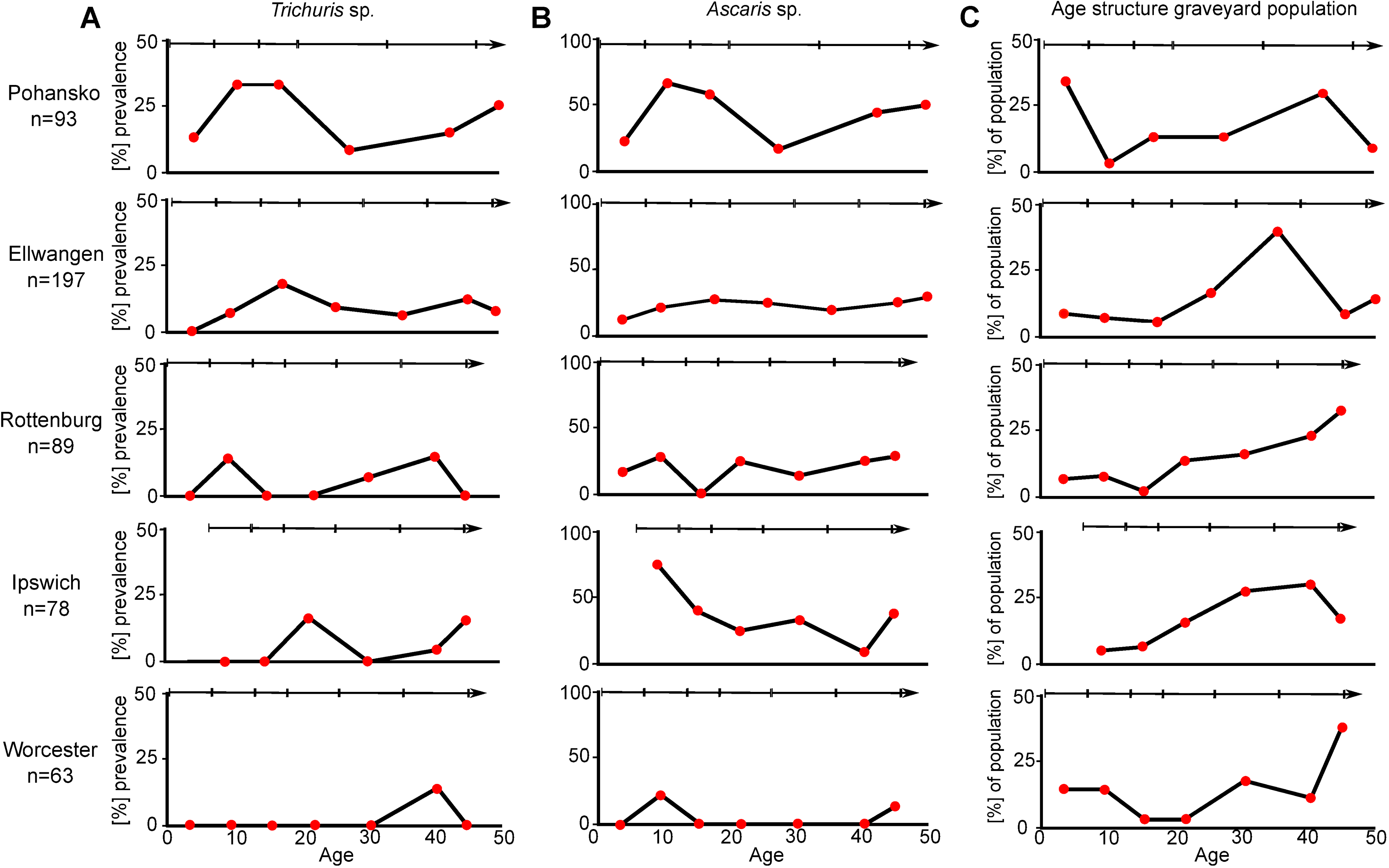

Supplement: S1 Fig — Age-associated prevalence for the common nematode helminths Trichuris (A) and Ascaris (B) was analysed within and across sites. The age structure (C) varied considerably across the sampled sites. (TIF) [file pntd.0008600.s001.tif]

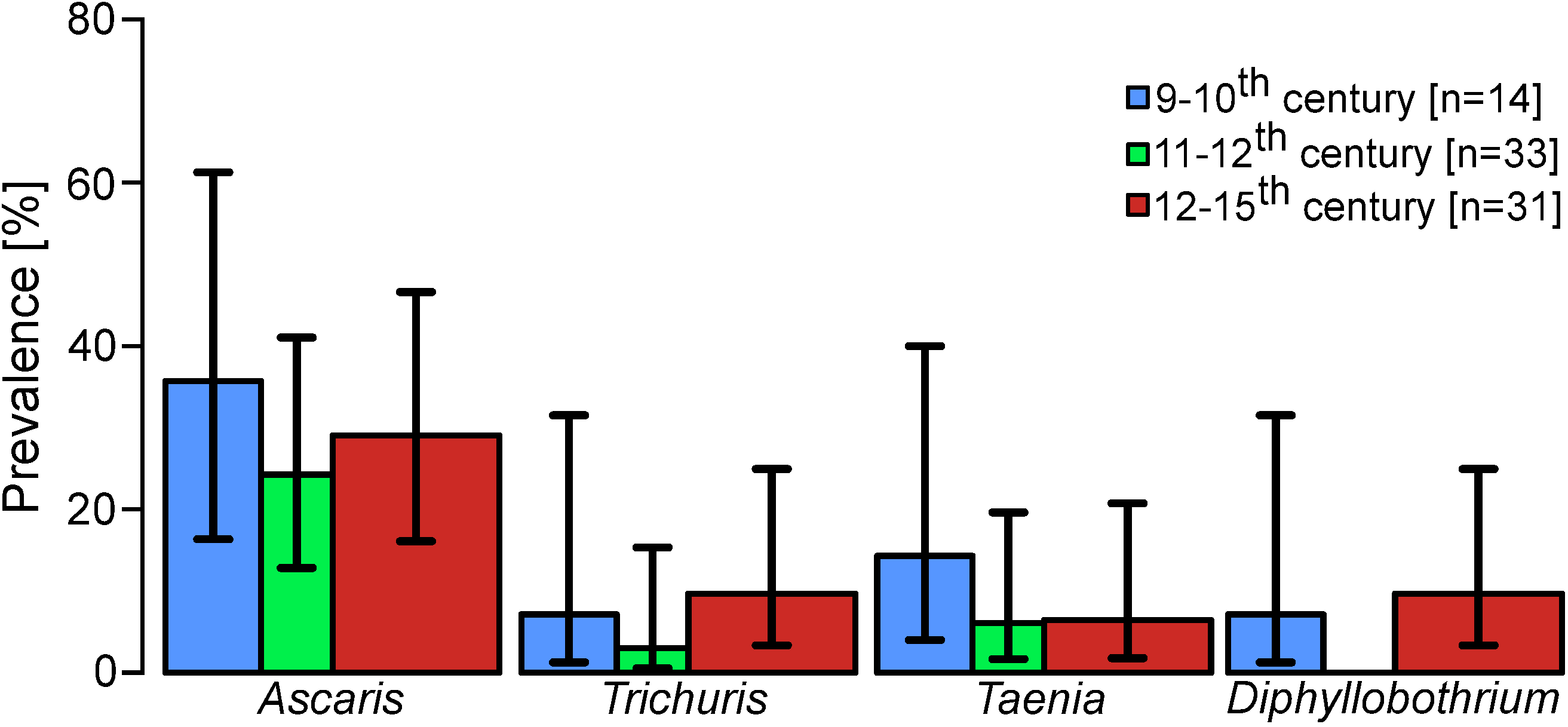

Supplement: S2 Fig — The prevalence rates for Ascaris, Trichuris, Taenia, and Diphyllobothrium infection in Ipswich were segregated according to 3 time periods: 9th-10th c, 11th-12th c and 12th-15thc. The number of samples in each time period is also identified. Bars represent the proportion of infected individuals and error bars represent 95% confidence intervals. (TIF) [file pntd.0008600.s002.tif]
