# Supplementary material for: Epidemiological insights from a large-scale investigation of intestinal helminths in Medieval Europe
Source: PLoS Negl Trop Dis. 2020 Aug 27;14(8):e0008600. doi: 10.1371/journal.pntd.0008600 (PMC7451528; doi:10.1371/journal.pntd.0008600)
Supplement: Attachment — Submitted filename: Response to reviewera.docx [file pntd.0008600.s003.docx]

**Response to reviewer’s comments**

We thank the reviewers for thoroughly considering the manuscript and for their positive remarks. Below we summarise the changes we have made in response to the comments. Any changes are also highlighted in one version of the manuscript. Our responses/explanations are in blue text below the reviewers comment.

**Methods**

Reviewer #1: If the authors were to further define their methods, then this paper would be an especially valuable source. How were the eggs concentrated for analysis? Were the slides examined without concentrating the eggs? Were the sediment samples collected from the sacra? Were control samples analyzed from the cemeteries? Why were the microscopic preparations discarded? Where are the initial samples now stored?

We have clarified the methods to answer these questions. We have added sections to the methods that covers the following.

Briefly, to avoid differential losses of material during processing microscopy was performed directly on a subsample of the rehydrated material. All sediment samples were taken from the sacral region of the remains. Although “control” (e.g. sediment from the skull) were not analysed for all graves this was performed for a subset of samples, we also analysed sediment taken from the subsurface and a similar depth to the skeletal level from some graveyards, we also sampled non-graveyard associated sediment. In all cases these “control” sediments contained no helminth eggs or parasite aDNA. To avoid potential cross-contamination the microscopy subsamples were discarded and not included in any part of the aDNA analysis. The samples for aDNA were sieved to remove large debris (pore sizes 1030 µm, 500 µm, 100 µm), centrifuged (400g for 10min), re-suspended in 1ml of TE buffer (Tris-HCl, Qiagen elution buffer) and disrupted using 1mm glass beads and a Beadbeater (BioSpec Inc, USA). The remainder of all primary samples and the aDNA samples are archived at the Department of Zoology, University of Oxford, with permission of the site archaeologists.
Reviewer #2: The methods are clearly defined, however there are located after the discussion section, it is necessary to move them after the results section. The study design is appropriate as well as the sample size.

We have moved the methods section to after the introduction, as indicated by the format guidelines.

**Results**

Reviewer #1: The results are well-organized and succinct
Reviewer #2: Results are clear and following the study design. The figures are sufficient but a couple of tables could help the results presentation and summary, for example the prevalence of helminths in the different study sites.

The prevalence rates for each helminth in each site are provided in the table associated with figure 1 similarly we provide a detailed table as part of fig 3 (with respect to prevalence in males and females).

**Discussion and conclusions**

Reviewer #1: The conclusions and discussion are adequate.
I think that basic scholarship could be improved by citing the following:
Camacho et a1 (2018) Recovering parasites from mummies and coprolites: an epidemiological approach. Parasites & Vectors (2018) 11:248
https://doi.org/10.1186/s13071-018-2729-4
This paper addresses and introduces a paleoepidemiological approach to archaeological parasitology.
We have inserted the reference with a short comment in the first paragraph of the Discussion. We have not gone into detail discussing the subtleties of analysing coprolites and mummies versus sacral sediment as we considered it inappropriate in the current context.
Leles et al (2010) A parasitological paradox: Why is ascarid infection so rare in the prehistoric Americas? J Archaeol Sci. 2010;37:1510–20.
This paper summarized all reports from Europe to assess the commonness of geohelminth infection compared to other prehistoric records and developed reasons for the high soil transmitted parasite experience in Europe.

Reference inserted in the first paragraph of the Discussion.
Replace Reinhard KJ. Parasitology as an Interpretive Tool in Archaeology. Am Antiquity. 644 1992;57(2):231-45
with Reinhard, K., 2017. Reestablishing rigor in archaeological parasitology. International journal of paleopathology, 19, pp.124-134. The 1992 reference is antiquated and the 2017 paper takes on issues that are current.

The 1992 reference has been replaced with 2017 reference.

Reviewer #2: The conclusions are supported by the data and analysis undertaken. Limitations are mentioned in some parts but not clearly expressed. In some parts, such as page 24 line 387, there is a repetition of results which is not needed.

We have removed the repetition of results that appeared in the discussion.

**Editorial and Data Presentation Modifications**

Reviewer #1: Here are optional suggestions for the manuscript.
“Prevalence”, in parasitology parlance, refers to the total number of individuals in a population are infected at a specific period of time, usually expressed as a percentage of the population. In the case of this paper, prevalence is the number/percentage of interments positive for infection evidence. I suggest that prevalence as used by the authors be defined in the Introduction.
A good point, as requested we have added text clarifying this in the last paragraph of the introduction.
Reviewer #2: Minor English revision is needed in the document

We have re-read the manuscript and made minor revisions.

**Summary and General Comments**
Reviewer #1: This paper could be published as is. It is a very significant, novel approach to assessing paleoepidemiology for Europe. It represents a monumental amount of work and innovative integration of standard microscopy and molecular biology. It established cemetery analysis as a statistically reliable manner of collecting hundreds of data points. Thus, based on this research, burial analysis takes its place alongside shaft feature analysis as key approach to defining infection across cultures, time and geography. It fits into an emerging realization that, for the Eurasian continent, Europe had a unique parasite pattern defined by geohelminths. Geographically, this pattern is not found in central Russia and westward. Therefore, Europe was an endemic area characterized by remarkably lax sanitation and hygiene controls.

Reviewer #2: In summary is well designed, implemented and presented. However, there are some statements that need further evidence or references for support, such as the one in lines 549-464. The study does provide with helpful epidemiological insights to intestinal helminths that could be useful in certain endemic regions.

We have added a small piece of text (and references) relating to our statement that reductions in STH endemicity were likely due to changes in WASH and related circumstances.
